# An Update on Myocarditis in Forensic Pathology

**DOI:** 10.3390/diagnostics14070760

**Published:** 2024-04-03

**Authors:** Jessica Falleti, Pasquale Orabona, Maurizio Municinò, Gianluca Castellaro, Giovanna Fusco, Gelsomina Mansueto

**Affiliations:** 1 Patology Section, Sant’Anna e San Sebastiano Hospital, 81100 Caserta, Italy; shabo1@inwind.it (J.F.); pasquale_orabona@yahoo.it (P.O.); 2 Forensic and Legal Medicine Center, San Giuliano Hospital, 80014 Naples, Italy; maurizio.municino@aslnapoli2nord.it; 3 University Department of Experimental Medicine—Forensic and Legal Medicine Unit, University of Campania “Luigi Vanvitelli”, 80138 Naples, Italy; gianluca.castellaro@gmail.com; 4 Experimental Zooprophylactic Institute of Southern Italy, 80055 Portici, Italy; giovanna.fusco@izsmportici.it; 5 University Department of Advanced Medical and Surgical Sciences, University of Campania “Luigi Vanvitelli”, 80138 Naples, Italy; 6 Department of Healthcare and Public Services—Forensic and Legal Medicine Unit, University of Campania “Luigi Vanvitelli”, 80138 Naples, Italy

**Keywords:** myocarditis, etiopathology, histopathology, classification, diagnosis

## Abstract

In forensic medicine, myocarditis is a complicated topic in the context of sudden death and medical malpractice. A good knowledge of the etiopathology, histopathology, and available literature are both indispensable and essential for the correct management and evaluation of the causal link. Some agents, which are rarely lethal for humans, are not necessarily related to death from myocarditis, even if an infection in other organs such as the gastrointestinal tract is documented. The diagnosis of the causes of death is often difficult and confusing. In some cases, the hypothetical diagnosis of myocarditis as the cause of death is formulated by deduction, causing error and misleading the correct temporal evaluation of pathological events. We reviewed the literature realizing that histomorphological data are scarce and often poorly documented. Only after COVID-19 have the histomorphological aspects of myocarditis been better documented. This is due to poor autopsy practice and poor accuracy in identifying the specific histotype of myocarditis with identification of the responsible agent. We believe that four points are essential for a better understanding and complete diagnosis of the disease: (1) clinical classification of myocarditis; (2) etiological classification of myocarditis; (3) pathophysiology of viral and bacterial infections with host response; and (4) histopathological diagnosis with precise identification of the histotype and pathogen. In the review we provide histological images from authoritative scientific references with the aim of providing useful information and food for thought to readers.

## 1. Introduction

In forensic medicine, the myocarditis is a complicated topic in the context of sudden death and medical malpractice. The guidelines for diagnosing and treating of myocarditis are a valuable aid [1,2], but compliance with diagnostic and therapeutic criteria often does not prevent death. In order to be useful in daily forensic legal medicine and forensic pathology activity, it is deemed appropriate to emphasize that a good knowledge of the etiopathology, histopathology, and literature available are indispensable for proper management and causal nexus. Some agents, which are rarely lethal for humans, are not necessarily related to death from myocarditis, even if an infection in other organs such as the gastrointestinal tract is documented. In fact, in some forensic cases, the hypothetical diagnosis of myocarditis as the cause of death is formulated by deduction, causing the incorrect temporal evaluation of events and management of the hypothesis of crime or medical malpractice. The review of the specific literature highlights that histological data are scarce and often poorly documented, and only after COVID-19 have many published histopathological images for the identification of viral myocarditis. This is due to the poor autopsy practice and the poor accuracy in identifying the specific histotype of myocarditis with the identification of the responsible agent. In fact, it often happens that in forensic practice we stop at identifying myocarditis generically as a cause of death without defining the responsible agent, forgetting that there are causes and concomitant causes. Therefore, it is essential for the correct understanding and complete diagnosis of myocarditis to know the clinical picture, etiological classification, and different pathogenesis based on the different causal agents with specific host response, and always request histopathological confirmation with a specific histotype. It is desirable to learn to use the molecular biology request on fresh samples when possible and on paraffin when the diagnostic hypothesis is not evident in order to provide evidence of the isolated pathogen. The performance of histological examination in all cases of myocarditis, especially in deaths, is a valid element for the good conduct of a judicial proceeding, but is also a documentary tool for future literature.

## 2. Materials and Methods

A comparison of literature data with histopathological data from autopsies and cardiac biopsies was performed for the diagnosis of myocarditis. A review of relevant literature relating to myocarditis was carried out by searching English language publications listed in PubMed using the following search terms: ‘myocarditis’, ‘autopsy’, ‘biopsy’, ‘histology’, and ‘death’. Articles included relevant retrospective studies, case reports, pathology cases and series, and review articles published between 1945–2024. 

## 3. Results

The search highlighted 7700 results from between 1945–2024, of which 2700 related to the COVID-19 pandemic (2020–2024). Only 853 prior to the COVID-19 period refer to histological data and only a very small part has reliable photographic documentation in compliance with histological classification and pathogenesis (Supplementary Material Table S1). 

## 4. Discussion

The first observation is that in the literature, the diagnosis of myocarditis is generic, clinical, without histological confirmation, and without identification of the causes. The second observation is that we are often unable to distinguish between bacterial, viral, and chemical/physical or hypersensitivity agents. The third observation is that, until 2020, there was a lack of autopsies and histopathological data. The fourth observation is that rearrangement must occur in order to correctly identify the histotype of myocarditis and the responsible agents in line with the development of technologies to correctly frame the causes of death.

### 4.1. Myocarditis—Clinical Classification

Myocarditis is an inflammatory involvement of the myocardium that can occur alone in the primary form or be part of systemic, immune, autoimmune, and infectious diseases, representing the secondary localization. It is characterized by heterogeneous etiology, variable often nonspecific clinical manifestations, and complex histopathology. The clinical presentation is characterized by a broad spectrum of symptoms, including chest pain resembling acute coronary syndrome, acute heart failure, cardiogenic shock, chronic heart failure, and conduction disturbances such as bradyarrhythmias or tachyarrhythmia. Less specific symptoms may include fever and abdominal pain, while in some cases, an unfortunate event with sudden cardiac death may occur in the absence of clear signs and symptoms. Although the outcome is favorable in half of the patients (50%), the other half may develop severe hemodynamic, electrical instability, acute heart failure, progressive dilated cardiomyopathy, or even sudden cardiac death [1,2,3]. 

The classification derives from clinical and radiological studies on humans while the histological data and the myocarditis dating derives mainly from experimental animal studies, as human tissue sampling are rare.

**Figure 1:**
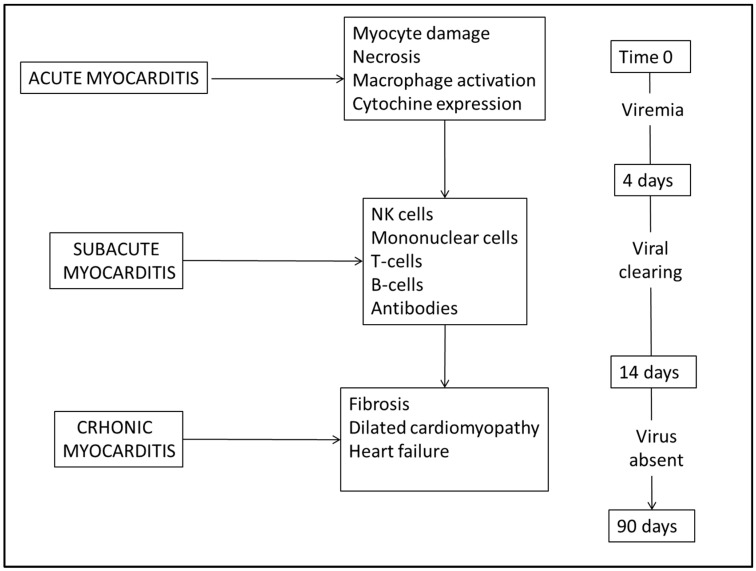
Dating of viral myocarditis with main cell types and chemical mediators involved.

Briefly, clinical studies identify the following forms. (1) Fulminant with acute heart failure in the two weeks following a viral infection. The most serious patients require mechanical hemodynamic support, and the outcome is fatal. (2) Acute with unclear evidence of the disease, systolic ventricular dysfunction and possible progression to a dilated cardiomyopathy. (3) Subacute with no evident symptoms but with the presence of cardiomyocytes damage despite the low viral load. (4) Active chronic with systolic ventricular dysfunction associated with chronic inflammation and frequent reactivations. (5) Persistent chronic clinically characterized by retrosternal pain, palpitations in the absence of significant ventricular dysfunction. In Figure 1 we schematize the dating of viral myocarditis consistently with experimental data [4] with the main cell types and chemical mediators involved, believing that it can be a valid starting point when it is necessary to verify the times between the onset of disease and death.

### 4.2. Myocarditis—Etiological Classification

Myocarditis is divided into infectious and non-infectious (Table 1). Viruses certainly represent the most common cause, but rarely can other infectious agents can be responsible [5].

Viruses, bacteria, parasites, fungi, and protozoa are responsible for infectious myocarditis. Bacteria include gram-negative microorganisms, aerobic and facultative intracellular bacilli of the Enterobacteriaceae family such as Salmonella, which rarely cause myocarditis but induce diagnostic doubt due to the absence of possible comparison with scarce histopathological data.

The causes of noninfectious myocarditis primarily include autoimmune conditions with cardiomyocyte damage from the activation of auto-immune mechanisms. In infective myocarditis, a cell-mediated and/or humoral inflammatory response with the release of cytokines, TNF, INF, interleukins, other molecules, and antibodies are responsible of cardiomyocytes damage. The variability of the cellular response and inflammatory mediators is influenced by specific agents, assuming pathognomonic characteristics are useful for diagnosis and targeted therapeutic treatment. The viral and bacterial/parasitic myocardial damages are produced by favoring one or both mechanisms—primary/direct and secondary damage in viral forms and in some parasitic forms; secondary damage in bacterial forms. Therefore, even when the clinical, radiological, and laboratory findings are indicative of an inflammatory process affecting the myocardium, the certainty of the type of infections, autoimmune or other causes, is exclusively histomorphological and is validated by isolation from cardiomyocytes of pathogen genome with molecular biology. 

To understand the mechanisms of infection, replication, and spread of the pathogen (and the consequential host response), it is necessary to take a step back and brush up on the pathophysiology of infections in general, independent of myocarditis. 

### 4.3. Pathophysiology of Viral and Bacterial Infections—Host Response

#### 4.3.1. Viral Infections

**Figure 2:**
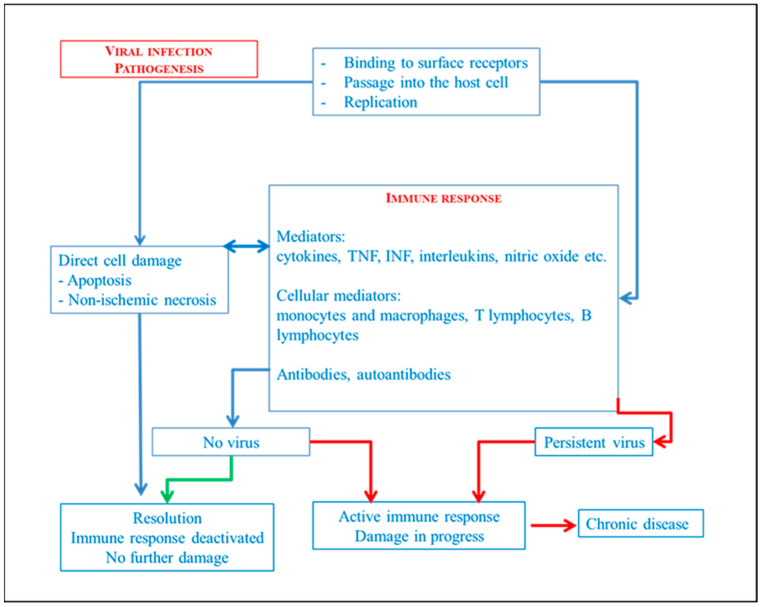
Simplification of viral infections pathogenesis.

**Figure 3:**
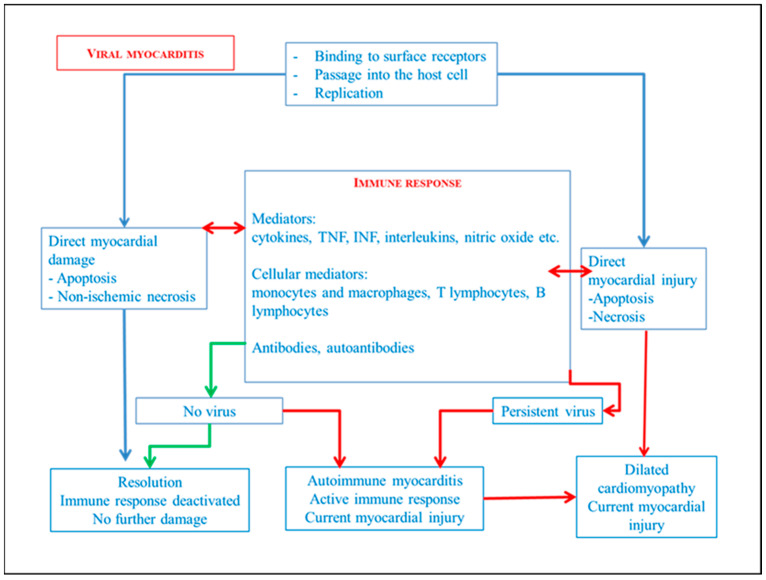
Simplification of viral myocarditis pathogenesis.

Viruses are obligate intracellular microorganisms. They enter cells by binding to surface receptors that vary from species-to-species and from cell-to-cell, and replicate using the host’s nucleic acid and protein synthesis system. Viruses can damage and kill the cell directly by inducing apoptosis or activating host cytocidal responses. In humans, the innate response is mediated by interferon, natural killer cells (NK), and macrophages. The virus directly stimulates the production of interferon by the infected cells, whose function is to inhibit viral replication in nearby cells. Natural killer cells (NK) are able to lyse cells infected by the virus recognizing a diversity of self on the cell surface. Macrophages eliminate virions from the intercellular spaces and from the vascular system. The specific antiviral adaptive response involves the combination of mechanisms mediated by monocytes, macrophages, T lymphocytes, B lymphocytes, and humoral antibody mechanisms [6,7,8,9]. Figure 2 briefly simplifies the mechanisms while Figure 3 transposes the main mechanism during viral myocarditis.

#### 4.3.2. Bacterial Infections

**Figure 4:**
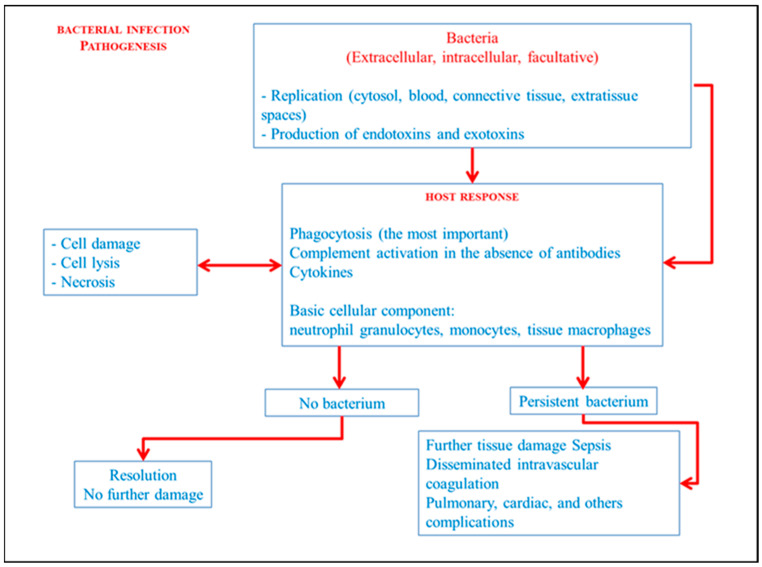
Simplification of bacterial infections pathogenesis.

**Figure 5:**
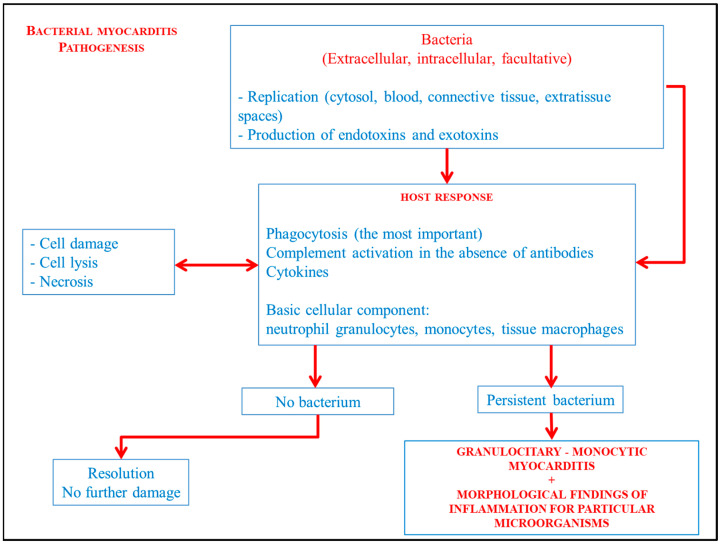
Simplification of bacterial myocarditis pathogenesis.

Bacteria defined as intracellular survive and replicate only in the cytoplasm and vacuoles of the host cell. Conversely, extracellular bacteria are able to replicate outside the cells in the blood, connective tissues, and extra-tissue spaces. Facultative intracellular bacteria have both capabilities. Gram-positive cocci, Gram-negative cocci, Gram-positive bacilli, Gram-negative bacilli with Enterobacteria, and Salmonella are examples of facultative bacteria. Extracellular bacteria cause disease through the induction of an inflammatory response and production of toxins with consequent destruction of the site of infection. Endotoxins and exotoxins, which are molecules and enzymes released by the bacteria and its lysis, are responsible for the destruction of tissues in the site of infection. Immune response against extracellular bacteria is aimed at eliminating the bacteria and neutralizing the effects of the toxins. Phagocytosis, cytokines and complement activation in the absence of antibodies represents the main mechanisms of natural immunity against extracellular bacteria. Therefore, the cellular component that participates most in the inflammatory response is basically represented by neutrophil granulocytes, monocytes, and tissue macrophages. For some bacteria, parasites, and fungi, the inflammation will be characterized by additional morphological findings such as granulomatous reactions or caseous reactions, which are the same in all localizations of the infection [6,7,8,9]. Figure 4 briefly simplifies the general bacterial infectious mechanisms while Figure 5 transposes the main mechanism it in the bacterial myocarditis.

The morphology of histological lesions during myocarditis reflects the etiopathogenetic mechanisms by identifying the defined and prevalent histotype.

### 4.4. Myocarditis—Histopathological Findings

A correct histopathological diagnosis of myocarditis must evaluate the presence or absence of cardiomyocytes necrosis, the type and quantity of inflammatory infiltrate, the presence or absence of fibrosis, and any perivasculitic aspects. Conventional histology highlights the basic characteristics, but the use of accessory methods can be very useful to confirm or better define the diagnosis with identification of the cause, the primitiveness of the disease, or the secondary localization. The immunohistochemistry/immunofluorescence to identify the specimens, among which the population of the T and B lymphocytes immunoglobulins, monocytes macrophages, granulocytic neutrophils, eosinophils, and basophils provides important information on the type of inflammatory process and the prevailing mechanisms. The histochemical stains are useful in doubtful cases to identify bacteria, fungi, and parasites present in macrophages activated in phagocytic activity or in cardiomyocytes. The molecular biology with the polymerase chain reaction method (PCR) on myocardial tissue can represent the discriminating proof between infectious and non-infectious myocarditis as well as proof between primary localization of the pathogen.

In the differential diagnosis between viral myocarditis and bacterial myocarditis, it should be considered that bacterial form is generally associated with endocarditis, while viral form is mainly localized in the myocardium. The early endocardial localization with the typical valvular vegetation compared to the late myocardial localization is explained by the particular ability of extracellular and facultative bacteria to spread and replicate in the blood. 

It is fundamental to underline that histology through identification of myocarditis histotype constitutes the only test capable of confirming a clinical or instrumental diagnosis of myocarditis. It is also the only test which can identify the infectious and non-infectious cause. In fact, cardiac biopsy is normally performed in subjects at risk of myocarditis in monitoring cardiac rejection in autoimmune forms [10].

According to the World Health Organization (WHO), the diagnosis and definition of the histotype of myocarditis must be performed in compliance with the Dallas, immunohistochemical, and immunological criteria. The morphological findings to be evaluated are inflammatory infiltrate ≥ 14 leukocytes/mm^2^ including up to 4 monocytes/mm^2^ with the presence of TCD3-positive lymphocytes ≥ 7 cells/mm^3^, presence or absence of myocytes degeneration, presence of ischemic and non-ischemic necrosis [1]. 

The cells involved in inflammation and the immune response perform specific functions. For example, monocytes, which are precursors to macrophages, act in the response against infectious agents but also in immune-mediated reactions by engulfing both infectious agents and residues of dead or damaged cells. They are therefore not specific unless they are identified in phagocytic activity when they contain in their abundant cytoplasm residues of specific pathogens rather than others. Neutrophils are often involved in bacterial infectious processes. Eosinophils are often involved in autoimmune reactions but are often observed histologically in other diseases and genetic pathologies. CD3+ T lymphocytes with the CD4+ and CD8+ subpopulations are often involved in viral infections and genetically determined autoimmune diseases. 

Therefore, the findings in the histological examination are generally indicative of different pathogens and pathogenesis. However, the correlation between histopathological patterns, clinical presentation and disease course in myocarditis is still largely unresolved, especially if we add particular patterns that are observed in other diseases such as genetic cardiomyopathies in which the inflammatory component can be confounding, but also in the inflammatory phase of the myocardial infarction. In the diagnostic algorithm, it is necessary to consider the Dallas criteria, which is the inflammation characteristic of viral, bacterial, parasitic infections, autoimmune reactions, but also specific types of inflammation which can occur in typical and atypical mycobacteriosis, aspergillosis, and other conditions. It should also be considered that bacterial infectious processes rarely involve the myocardium exclusively, and often initially affect the parietal and valvular endocardium. Furthermore, other diseases must be considered, including cardiomyopathies and some lymphoid infiltrations of a neoplastic nature.

Pathologists therefore identify some main histological patterns of myocarditis: lymphocytic myocarditis (LM); lymphohistiocytic myocarditis (LHM); neutrophilic myocarditis (NM); eosinophilic myocarditis (EM); giant cells myocarditis (GCM); granulomatous myocarditis (GM); myocarditis with vasculitis microvascular inflammation (MVMI); and toxic myocarditis (TM).

#### 4.4.1. Lymphocytic Myocarditis

The Lymphocytic myocarditis is the most common virus-associated histotype with inflammatory infiltrate typically consisting of T-lymphocyte component and variable monocyte-macrophage representation. A small amount of neutrophil granulocytes may be present, usually related to the extent of myocyte damage. Rare plasma cells and eosinophils can be seen. If neutrophilic or eosinophilic granulocytes are more represented, it is necessary to consider alternative diagnoses of myocarditis with different etiopathogenesis [11,12]. Myocyte necrosis is observed in the active phase of lymphocytic myocarditis, although it is less relevant than inflammation, while it is more extensive in the advanced phases. Necrosis can be localized and sporadic in subacute or healing lymphocytic myocarditis, although it may be absent in cases that are treated therapeutically. It is therefore necessary to consider not only lymphocyte inflammation, but also the possible presence of macrophages when necrosis triggers phagocytosis of damaged structures and the fibrotic outcome of healing. 

The main etiology of lymphocytic myocarditis is viral, but we must not forget that secondary forms caused by severe autoimmunity also exist [5,11,12,13,14,15,16,17,18,19]. In these forms, myocyte necrosis tends to be less evident and often presents with lymphocytic vasculitis of small vessels or chronic remodeling of small vessels. In an interesting review, Leone et al. report some microscopic documentations which show that in lymphocytic myocarditis the presence of a diffuse amount of T lymphocytes with a minor component of macrophages together with edema and myocyte damage and rare neutrophils attracted by myocyte necrosis. Furthermore, the same authors report that in healing myocarditis, the lymphocytic infiltrate is reduced and mainly localized within the fibrous mesenchymal reparative tissue with a more evident share of CD68+ macrophages [5]. The review represents one of the few scientific papers documenting the morphological findings of myocarditis.

#### 4.4.2. Lympho-Histiocytic Myocarditis

Lympho-histiocytic myocarditis is characterized by lymphocytes mixed with a monocyte-macrophage component. Monocytes and macrophages are numerous and can represent the prevalent cell population. The histological pattern may be related to recovery of the lymphocytic form, unusual forms of viral myocarditis, and autoimmune diseases in which macrophages play a major role in antibody-mediated inflammation [20,21,22]. Finally, for a correct differential diagnosis, we note that chronic viral myocarditis has a histological pattern with a rich histiocytic component. Indeed, the Kindermann study group also reports examples of acute phase viral myocarditis with myocyte necrosis and mononuclear infiltrate that includes CD3+ T lymphocytes and B lymphocytes, and examples of chronic viral myocarditis with CD68+ macrophage infiltrate and areas of fibrosis. In similar cases, the detection of viral nucleic acids in endothelial cells and of ribonucleic acid in myocytes in chronic myocarditis reinforces the documented presence of histiocytes in viral infections [5,11]. 

#### 4.4.3. Neutrophilic Myocarditis

Neutrophilic myocarditis is a rare histotype related to bacterial infections associated with pancarditis, and is the myocardial involvement most frequently due to hematogenous dissemination in septicemia. Neutrophils predominate and may be irregularly distributed or form micro-abscesses around damaged myocytes and more significant areas of necrosis, however, these findings can also be found in fungal myocarditis, occasionally coexisting with granulomas and giant cells. In rare cases, granulomatous inflammation and lympho-histiocytic infiltrates with a variable degree of neutrophils may be present. Therefore, it is important to pay attention to possible localizations of diseases such as tuberculosis, leptospirosis or even, albeit rare, other granulomatous diseases [23,24,25]. 

The typical neutrophil-rich infiltrate that is present in Gram-negative bacterial infections and Salmonella infection undoubtedly deserves special attention. The typhoid and paratyphoid salmonella are responsible for systemic diseases and rarely can also cause secondary myocarditis. Non-typhoid forms have a propensity to cause gastrointestinal symptoms, and being able to replicate also in the bloodstream, have the ability to spread hematogenously, causing rare extraintestinal complications mainly represented by pneumonia, osteomyelitis, and central nervous system infections. We must take into account for a diagnosis of myocarditis in the cases mentioned that cardiac complications from salmonellosis are extremely rare and represented by pericarditis, endocarditis, and myocarditis (more often simultaneously and rarely by myocarditis alone). We must also consider that the same complications can occur in immunocompromised individuals or with pre-existing vasculopathies and valvulopathies which are predisposing factors for the development of bacterial endocarditis. The diagnosis of Salmonella infection is usually confirmed visualizing the pathogen in the tissue in the tissue. The integration of the morphological data on hematoxylin and eosin stain with histochemical and immunohistochemical stains as well as with PCR are undoubtedly useful when the diagnostic doubt is persistent. In the literature, there are few microphotographic documentations of the neutrophil granulocyte pattern in the heart of subjects who died of myocarditis in the course of salmonellosis, and it is superimposable to the histological pattern that is generally observed in the primitive intestinal localizations. This derives from the ability to self-limit infections without leading to death and the consequent poor autopsy documentation. Instead, there are autopsy studies on animals that support the pathophysiology of bacterial infections in humans by validating the concept that bacterial inflammation in secondary sites with sepsis is similar to that present in the primary sites of infection. For example, most Enterobacteria are responsible for necrotizing enterocolitis with neutrophils, mixed lymphocytic and macrophages, ulcerations of the intestinal mucosa, edema, congestion, hemorrhages, and thrombosis of small vessels as aspects of the activation of the complement pathway [26,27,28,29,30,31,32,33,34,35]. In these cases, the microscopic observation of the heart cannot ignore the microscopic observation of the intestine compared with the morphology.

#### 4.4.4. Eosinophilic Myocarditis

The eosinophilic myocarditis is a histotype associated with a broad spectrum of diseases and conditions. Hypersensitivity reactions, immune-mediated diseases, idiopathic hypereosinophilic syndrome, non-hematological malignancies, parasitic infections, drugs, vaccines, and others may have overlapping aspects. The common denominator is the eosinophilic infiltrate, which can represent the major inflammatory component in relation to the underlying disease or the type of stimulus, or it can be part of a complex condition of mixed inflammation with lymphocytes, macrophages, plasma cells, giant cells, and granulomas [5,17,36,37]. In these cases, the basic histology must be supplemented by special stains and sometimes also by immunofluorescence for antibody evaluation. A complex diagnosis can become simple if placed in the right context.

#### 4.4.5. Giant Cell Myocarditis

The giant cell myocarditis is a distinct clinical-pathological form, often with fulminant prognosis. This type of myocarditis is believed to be autoimmune in nature, and is characterized by mixed inflammatory infiltrate consisting mainly of macrophages, multinucleated cells, and to a lesser extent, lymphocyte, eosinophils, and plasma cells. As the disease progresses to chronicity, inflammation is less widespread and severe, giant cells become rare, myocyte damage becomes focal, and reparative fibrosis appears [5,13,17,38,39,40,41,42]. Reparative fibrosis can create the diagnostic doubt of an infarct outcome.

#### 4.4.6. Granulomatous Myocarditis

The Myocarditis with granulomas is characterized by the presence of granulomas. Cardiac sarcoidosis, which is usually part of multiorgan system disease, although primary cardiac sarcoidosis has also been described, certainly deserves attention in this chapter. The histological findings are characterized by non-necrotizing, non-confluent epithelioid giant cell granulomas surrounded by T lymphocytes. In addition, foci of exclusively lymphocytic myocarditis can often be observed while in late lesions, and sclerotic areas with confluent granulomas are present [43,44,45]. In tuberculosis, atypical mycobacteriosis, and others parasitic/fungal disease, histology shows patterns with different granulomas. A necrotizing, suppurative, or caseous granulomas in myocardial sampling may aid in the differential diagnosis, as occurs for other sites However, histochemical stains such as Ziehl Neelsen, PAS, Grocott, Giemsa, and other stains are useful to support the diagnosis and distinguish between acid-fast bacilli, fungi, parasites, etc. It seems obvious that the histology must be interpreted in the broader clinical context. 

#### 4.4.7. Myocarditis with Vasculitis and Microvascular Inflammation

Myocarditis with vasculitis and microvascular inflammation is generally associated with systemic autoimmune disorders. The histological findings may resemble those observed in lymphocytic, lymphohistiocytic, and eosinophilic myocarditis. The diagnosis is complex and requires a lot of clinical and laboratory information [21,22,23,24].

#### 4.4.8. Toxic Myocarditis

Toxic myocarditis is caused by chemical agents. The drugs and illicit substances can damage the myocardium directly and/or with hypersensitivity reactions and through the release of catecholamines. The histology is often variable and nonspecific. However, the initial stage shows main features represented by micro-foci of necrotic myocytes, damaged with an eosinophilic appearance, and with evidence of contraction bands, while the later stage has more clear features of myocarditis. In these cases, the inflammatory infiltrate can be mixed and refers to hypersensitivity infiltrates and lymphohistiocytic forms [46,47,48,49]. The myocardial damage is significant and can take on spotty myocardial infarction characteristics such as in cocaine abuse. The presence of spotty areas or fibrous replacement helps to understand the chronicity of the intake. In these cases, rather than the inflammatory infiltrate, attention must be paid to the type of myocyte damage and ischemic infarct and its phases. 

In the most recent period, during and after the COVID-19 pandemic, the literature has reported myocarditis secondary to both the virus and the administration of vaccines. Histology showed characteristics of predominantly lymphocytic and lymphohistiocytic viral myocarditis during SARS-CoV19 infection, while it showed characteristics similar to the toxic form in the presence of vaccine adverse events with a peculiar appearance of necrotic myocytes, contraction bands, and lymphomonocytic infiltrate. Kiblboeck D et al. provided interesting histological and immunohistochemical images of acute post-vaccination myocarditis documenting the presence of viral mRNA and the presence of CD3+ T lymphocytes and CD68+ macrophages. Choi et al. reported the prevalent presence of CD68+ histiocytes, and the presence of CD4+ lymphocytes with associated scattered myocyte necrosis [5,50,51,52,53,54,55,56,57,58].

## 5. Conclusions

From the brief scientific excursus, it emerges that the histological documentation of myocarditis is still scarce, especially in the forensic literature prior to the COVID-19 period. The differential diagnosis between myocarditis and other cardiac diseases, in which inflammatory infiltrate is associated, is still complex, especially when questions are misleading. Many morphological findings can generate confusion in the eyes of the inexperienced because they are common to many diseases. In some cases, however, the “fall in love” with the initial hypothesis of death from myocarditis, built on the circumstantial data of infection, is misleading and leads to an incorrect evaluation of the events. The basic pathophysiology and histopathological classification with myocarditis histotype provides the first and irreplaceable tools for reasoning about autopsy findings. Histochemical and immunohistochemical techniques can support histological diagnosis and molecular biology can be extremely useful to define with reasonable certainty the exact cause of myocarditis through the isolation of the pathogenic genome in cases of infectious myocarditis. In the near future, it is desirable to improve autopsies by always including histological examinations and accompanying it with technologies and molecular biology in order to achieve standardized protocols that can be used in forensic practice. This would be of great help to avoid the unusual definition of myocarditis, which alone is not able to provide certainty about the precise cause of death. In more complex cases, it would be ideal to be able to establish with certainty that death occurred due to primary, secondary, or concomitant myocarditis with a different systemic infectious process. Only by defining the type of myocarditis and identifying the responsible pathogen in the cause of death will it be possible to improve our contribution. 

## Figures and Tables

**Table 1:** Myocarditis—etiological classification.

**Infectious Myocarditis**
**Viruses**
DNA viruses: Adenoviruses, Erythroviruses (Parvovirus B19), Hepatitis B virus, Herpes viruses (Herpes Simplex virus type-1 and type-2, Human Herpesvirus-type {6}, Cytomegalovirus, Epstein-Barr virus, Varicella-Zoster virus), Rabies virus, Variola virus, Vaccinia virus RNA viruses: Chikungunya virus, Enteroviruses (Coxsackieviruses A/B, Echoviruses), Hepatitis C virus, Human Immunodeficiency virus, Influenza A/B viruses, Measles virus, Mumps virus, Polioviruses, Rabies virus, Respiratory syncytial virus, Rubella virus, Coronavirus (SARS, MERS). Dengue virus, Junin virus, Lassa fever virus, Yellow fever virus
**Bacteria**
Brucella, Chlamydia, Clostidrium, Corynebacterium diphtheria, Haemophilus influenzae, Gonococcus, Legionella spp, Meningococcus, Mycobacteria, Mycoplasma pneumoniae, Pneumococcus, Salmonella, Staphylococcus, Streptococci, Vibrio cholera
**Protozoa**
Entamoeba histolytica, Leishmania, Plasmodium falciparum Trypanosoma cruzi (Chagas disease), Toxoplasma gondii Actinomyces, Aspergillus, Blastomyces, Candida, Coccidioides, Cryptococcus, Histoplasma, Mucormycoses, Nocardia, Sporothrix schenckii
**Spirochaete**
Borrelia burgdorferi (Lyme disease), Leptospira (Weil disease), Treponema pallidum
**Parasites and Rickettsia (very rare)**
Echinococcus granulosus, Schistosoma, Taenia solium, Toxocara canis, Trichinella spiralis, Coxiella burnetii (Q fever), Rickettsia rickettsii (Rocky Mountain spotted fever)
**Non-Infectious Myocarditis**
**Immune autoimmune**
Autoantigens: infectious-negative lymphocytic or giant cell myocarditis (post-infectious or auto-immune inflammatory processes) Associated with immune-mediated or autoimmune/autoinflammatory diseases: Behçet’s disease, Churg-Strauss syndrome, coeliac disease, inflammatory bowel disease (Crohn’s disease, ulcerative colitis), inflammatory myopathies (dermatomyositis, polimyositis), Kawasaki’s disease, sarcoidosis, systemic sclerosis, systemic lupus erythematosus, rheumatic heart disease (rheumatic fever), rheumatoid arthritis, juvenile idiopathic arthritis, ANCA associated vasculitis, non-antibody associated vasculitis including giant cell and Takayasu arteritis Alloantigens: heart transplant rejection Allergens: smallpox and tetanus toxoid vaccinations
**Drugs/toxic substances**
Allergic/hypersensitivity reaction (HSM) Antiphlogistics: mesalamine, phenylbutazone Psychiatric medications: benzodiazepines, clozapine, lithium, tricyclic antidepressants Antibiotics: ampicillin, azithromycin, cephalosporins ciprofloxacin, isoniazid, penicillin, sulphonamides, tetracyclines Miscellaneous: adalimumab, colchicine, thiazide diuretics, methyldopa, dobutamine, lidocaine, metoprolol, phenytoin Direct toxic effects (toxic myocarditis) Antineoplastic drugs: amsacrine, anthracyclines cyclophosphamide, 5-fluorouracil, imatimib mesylate, interferon-alpha, interleukin-2, mitomycin C, mitoxantrone, tyrosine kinase inhibitors (e.g., trastuzumab, anti-HER2/neu) Miscellaneous: amphetamine-derived compounds, antimony compounds, antiretroviral agents, catecholamines (dopamine, dobutamine, norepinephrine, epinephrine), chloramphenicol, chloroquine, hydroxychloroquine, dopamine agonists, emetine, ephedrine lithium, methysergide phenothiazines, phenylpropanolamine, serotonin-derived compounds (fenfluramine/phentermine, appetite suppressants), phenytoin, tricyclic antidepressants, zidovudin Drugs of abuse Alcohol, amphetamines and related compounds (methamphetamines, ecstasy), anabolic steroids, cocaine, opiate overdose
**Others**
Heavy metals: copper, iron, lead, arsenicals Physical agents: radiation Scorpion-bee-wasp stings, snake/spider bite

## Supplementary Materials

The following supporting information can be downloaded at: https://www.mdpi.com/article/10.3390/diagnostics14070760/s1.
