# Supplementary material for: An Update on Myocarditis in Forensic Pathology
Source: Diagnostics (Basel). 2024 Apr 3;14(7):760. doi: 10.3390/diagnostics14070760 (PMC11011922; doi:10.3390/diagnostics14070760)
Supplement: Supplementary file 1 [file diagnostics-14-00760-s001.zip › diagnostics-2900477-supplementary.pdf]

Supplementary material Table S1

LM	CASES	HISTOPATHOLOGY	IMAGE LINK
Leone O, et al. Virchows Arch. 2019 (5)	45-year male<br>40-year female<br>20-year male<br>30-year female	-Myocyte damage<br>T-lymphocytes with a minor component of macrophages; neutrophils are attracted by myocyte necrosis<br><br>- Lymphocytic infiltrate is limited but is anyway associated with myocyte damage<br><br>- Control biopsy in after previous histologic diagnosis of multifocal lymphocytic myocarditis. Lymphocytic infliltrate is reduced and mainly localized within the mesenchymal/fibrous reparative tissue<br><br>-Immunohistochemistry: CD3+ CD68+	https://media.springernature.com/full/springer-static/image/art%3A10.1007%2Fs00428-019-02615-8/MediaObjects/428_2019_2615_Fig1_HTML.jpg?as=webp
Kindermann I, et al. J Am Coll Cardiol. 2012 (11)	Patients with acute myocarditis	-Necrotic myocytes<br>-Mononuclear cell infiltrates<br>-Immunohistochemistry: CD3+	https://ars.els-cdn.com/content/image/1-s2.0-S0735109711052004-gr4.jpg
Caforio AL, et al. Eur Heart J. 2007 (13)	EMB from 174 subjects with arrhythmias and heart transplantation.	-Myocyte necrosis<br>-Fibrosis<br><br>-162 EMB: lymphocytes infiltrate<br>-5 EMB: giant cells infiltrate<br>-7 EMB: others misted infiltrate<br><br>-PCR analysis for viruses was performed	https://oup.silverchair-cdn.com/oup/backfile/Content_public/Journal/eurheartj/28/11/10.1093_eurheartj_ehm076/2/ehm07601.gif?Expires=1697016563&Signature=1grS0gGlcmtcJYuxiWhJ9ZqeabjP8D7OkVYEo-GuWllnDYb5gX8oQFbAZOVrKkWeAtiDIWNAsbzhsU0VZad8TyrE8pKD3ApbhO7bdRyvulKKHDI9BtHnp-QyY5POvwY5WbK7xtdvXz2uYo3phqerIJUySNKAIEJfHtnCrqOtD9-92gXgVc~kWg8bqkqiUq1ZEYcECKJc-

			YKuyIbxzBI3omqnKwaar7YG1ICwyzd GfFwv8STYWjkztkx6zzhfAKecOhnnsC xmbYWijthyYo~zyomri5Sh4hXrYPUI M5eDMCmaaT99OfXUc27SgXtBOQZt eO4Cb54a9ug6EsQskyYHUQ__&Key-Pair-Id=APKAIE5G5CRDK6RD3PGA
Mahler L, McCleskey B. Am J Forensic Med Pathol. 2022 (16)	2-year-old African American boy	-Myocytolysis<br>- Karyorrhectic debris<br>-Lymphohistiocytic infiltrate<br>-Lymphocytic infiltrate<br><br>-Myocarditis likely viral in origin<br><br>-PCR for virus negative	https://www.ncbi.nlm.nih.gov/pmc/articles/PMC9076121/bin/fmp-43-e12-g003.jpg
Kanaoka K, et al. Circulation 2022 (17)	EMB of 215 patients	- Myocytes damage<br>- 162 LM with lymphocytic infiltrate<br>- 42 EM with eosinophilic infiltrate<br>- 11 GCM with giant cell infiltrate	https://www.ahajournals.org/reader/content/184510a048b/10.1161/CIRCULATIONAHA.121.058869/format/epub/EPUB/graphic/circulationaha.121.058869.fig03.jpg?hmac=1693997579-CroByceU73XB44vh0kimzQ5OcO9BSPSfyZ5KJi86eak%3D
Kawada JI, et al. J Cardiol. 2021 (18)	Five pediatric patients	- LM was observed in all patients except in patient 5.<br>- CD3+ CD68+<br><br>-Similar levels of CD4+ and CD8+ T cells were observed in patient 1, while CD8+ cells were predominant in patients 2, 3, and 4.	https://ars.els-cdn.com/content/image/1-s2.0-S0914508720302896-gr2.jpg<br><br>https://ars.els-cdn.com/content/image/1-s2.0-S0914508720302896-gr3.jpg<br>https://ars.els-cdn.com/content/image/1-s2.0-S0914508720302896-gr1.jpg

		-Conversely, high infiltration of CD68+ cells were seen in patients 2 and 4. CD20+ and CD138+ cells were identified in patient 3<br><br>No significant virus-derived reads were detected	
Huang Z, et al. J Int Med Res. 2022 (19)	63-year-old woman	-Lymphohistiocytic infiltrate<br><br>-Immunohistochemistry: CD3+ CD68+	https://www.ncbi.nlm.nih.gov/pmc/articles/PMC9251992/bin/10.1177_03000605221108933-fig4.jpg
**LHM**			
Leone O, et al. Virchows Arch. 2019 (5)	17-year-old male with juvenile idiopathic arthritis	-Active multifocal myocarditis<br>-Macrophages mixed with fibrin and platelets, and minor component of granulocytes and T lymphocytes<br>-Microthrombi and granulocytes were also present in some small vessels.<br>-Immunohistochemistry: CD68+<br><br>-PCR for viral genomes on myocardial tissue and blood was negative as were other laboratory tests for serum immunologic assessment and bacterial or viral infections	https://media.springernature.com/full/springer-static/image/art%3A10.1007%2Fs00428-019-02615-8/MediaObjects/428_2019_2615_Fig6_HTML.png?as=webp
Kindermann I, et al. J Am Coll Cardiol. 2012 (11)	Patients with chronic myocarditis	-CD68+ cells are mainly present in areas with fibrosis<br><br>-In situ hybridization reveals PVB19 nucleic acid in endothelial cells of an arteriole in a	https://ars.els-cdn.com/content/image/1-s2.0-S0735109711052004-gr4.jpg

		patient with chronic myocarditis, whereas enterovirus ribonucleic acid is detected in several myocytes	
Cooper LT Jr. N Engl J Med. 2009 (12)	patient with acute myocarditis without other specific indications	-Myocyte damage<br>- Immunohistochemistry: CD3+ CD68+	https://www.ncbi.nlm.nih.gov/pmc/articles/PMC5814110/bin/nihms940711f1.jpg
**NM**			
Somers GR, et al. Arch Pathol Lab Med. 2005 (25)	Five drownings	-Occasional neutrophils foci of myocyte necrosis -Lymphocytes infiltrate	https://allen.silverchair-cdn.com/allen/content_public/journal/aplm/129/2/10.5858_2005-129-205-aodami/1/i1543-2165-129-2-205-f01.jpeg?Expires=1697106412&Signature=Im1V2MKPWNCnUIsxx9r8YCzVVaaSJKMW69cOC8oIE0744IOoJQDjar-FEvpAy2Pkq4F4shpcADBehgS-G63H4piw-7gU9ZeSaob4taiYPR00NdhNsPjol2sj82~cyFXA55QuUdwWM4t0TdI~E9vjtSH~oGJtcZPalnYdoSaTqpIFHSGLc1LTD~Ok4i5iK7OfRIG7DDcSlQNJWV-szLJPQ-8fQ0D62uRBcRLOQbNbN3iSrIb7pTPrtZQS5sLWAvlADVDM1bd5M~W~aJoNK5WaWaUDQUxEK1cBMDGRGFQ7sHehrTDWybk2hm2EOvxQVPwohA4dRhLp2y~ceeCQy0xkcQ__&Key-Pair-Id=APKAIE5G5CRDK6RD3PGA
Hiraiwa H, et al. J Cardiol Cases 2022 (26)	2-year-old man with streptococcal pharyngitis	- Micro-abscesses<br>- Neutrophilic granulocytes<br>- Gram stain + indicating phagocytosis of streptococci by neutrophils	https://www.ncbi.nlm.nih.gov/pmc/articles/PMC9214811/bin/gr2.jpg

Shilkin KB. Postgrad Med J. 1969 (27)	A 61-year-old male	Diffuse or focal infiltration of polymorphonuclear cells	https://www.ncbi.nlm.nih.gov/pmc/articles/PMC2466746/bin/postmedj00361-0043-a.jpg<br><br>https://www.ncbi.nlm.nih.gov/pmc/articles/PMC2466746/bin/postmedj00361-0043-b.jpg
Oka K, et al. Virchows Arch. 2005 (35)	42-year-old male<br>39-year-old male<br><br>Enterovirus	- Myocytolysis<br>-Patchy necrosis<br>- Neutrophilic abscess<br>-Infiltration of lymphocytes<br>- Cell-debris<br>- Macrophages	https://media.springernature.com/full/springer-static/image/art%3A10.1007%2Fs00428-004-1173-3/MediaObjects/s00428-004-1173-3fhb1.jpg?as=webp<br>https://media.springernature.com/full/springer-static/image/art%3A10.1007%2Fs00428-004-1173-3/MediaObjects/s00428-004-1173-3fhb2.jpg?as=webp
**EM**			
Leone O, et al. Virchows Arch. 2019 (5)	43-year-old male with primary hypereosinophilic syndrome	Eosinophils degranulated or not, together with some lymphocytes and macrophages and initial thrombotic deposition on the endocardium surface	https://media.springernature.com/full/springer-static/image/art%3A10.1007%2Fs00428-019-02615-8/MediaObjects/428_2019_2615_Fig3_HTML.jpg?as=webp
Leone O, et al. Virchows Arch. 2019 (5)	28-year-old male in therapy with mesalamine for recurrent ulcerative colitis	- Multifocal myocyte damage<br>-Microgranulomas with macrophages, eosinophils, neutrophils, lymphocytes<br><br>Immunohistochemistry CD68 + CD15+ CD3+	https://media.springernature.com/full/springer-static/image/art%3A10.1007%2Fs00428-019-02615-8/MediaObjects/428_2019_2615_Fig4_HTML.png?as=webp
Kanaoka K, et al. Circulation. 2022 (17)	EMB specimens from 215 patients	- Myocytes damage<br>- 162 LM with Lymphocytic infiltrate<br>- 42 EM with eosinophilic infiltrate	https://www.ahajournals.org/reader/content/184510a048b/10.1161/CIRCULATIO

		- 11 GCM with giant cell infiltrate	NAHA.121.058869/format/epub/EPUB/graphic/circulationaha.121.058869.fig03.jpg?hmac=1693997579-CroByceU73XB44vh0kimzQ5OcO9BSPSfyZ5KJi86eak%3D
**GCM**			
Leone O, et al. Virchows Arch. 2019 (5)	34-year male	-Widespread and Mixed Inflammation: macrophages, lymphocytes, variable component of eosinophils and plasma cells and a substantial number of scattered multinucleated giant cells.<br>-Significant myocyte damage<br>-No clearly formed granulomas	https://media.springernature.com/full/springer-static/image/art%3A10.1007%2Fs00428-019-02615-8/MediaObjects/428_2019_2615_Fig2_HTML.jpg?as=webp
Caforio AL, et al. Eur Heart J. 2007 (13)	EMB from 174 subjects (arrhythmias and heart transplantation with suspicion of myocarditis)	-Myocyte necrosis<br>-Fibrosis<br><br>-162 EMB: lymphocytes infiltrate<br>-5 EMB: giant cells infiltrate<br>-7 EMB: others misted infiltrate<br><br>-PCR analysis for viruses was performed	https://oup.silverchair-cdn.com/oup/backfile/Content_public/Journal/eurheartj/28/11/10.1093_eurheartj_ehm076/2/ehm07601.gif?Expires=1697016563&Signature=1grS0gGlcmtcJYuxiWhJ9ZqeabjP8D7OkVYEo-GuWllnDYb5gX8oQFbAZOVrKkWeAtiDIWNAsbzhsU0VZad8TyrE8pKD3ApbhO7bdRyvulKKHDI9BtHnp-QyY5POvwY5WbK7xtdvXz2uYo3phqerIJUySNKAIEJfHtnCrqOtD9-92gXgVc~kWg8bqkqiUq1ZEYcECKJc-YKuyIbxzBI3omqnKwaar7YG1ICwyzdGfFwv8STYWjkztkx6zzhfAKecOhnnsCxmbYWijthyYo~zyomri5Sh4hXrYPUIM5eDMCmaaT99OfXUc27SgXtBOQZteO4Cb54a9ug6EsQskyYHUQ__&Key-Pair-Id=APKAIE5G5CRDK6RD3PGA

Kanaoka K, et al. Circulation. 2022 (17)	EMB specimens from 215 patients	- Myocytes damage<br>- 162 LM with Lymphocytic infiltrate<br>- 42 EM with eosinophilic infiltrate<br>- 11 GCM with giant cell infiltrate	https://www.ahajournals.org/reader/content/184510a048b/10.1161/CIRCULATIONAHA.121.058869/format/epub/EPUB/graphic/circulationaha.121.058869.fig03.jpg?hmac=1693997579-CroByceU73XB44vh0kimzQ5OcO9BSPSfyZ5KJi86eak%3D
Larsen BT, et al. Circulation. 2013 (42)	Six patients	- cardiomyocyte necrosis and cardiomyocyte hypertrophy in all cases<br>-Giant cells, histiocites, T- and B- lymphocytes<br><br>-Immunohistochemistry: prevalent CD3+ with scattered CD20+ and numerous CD68+ cells	https://www.ahajournals.org/cms/asset/aab95466-3982-4d1c-87b6-c2d085950430/39fig03.jpg<br>https://www.ahajournals.org/cms/asset/1e1f3d2c-a6f8-49cc-a31a-61a2afdd8e47/39fig04.jpg
**CS**			
Leone O, et al. Virchows Arch. 2019 (5)	-47-year female with isolated cardiac sarcoidosis<br>- 32-year-old female with systemic sarcoidosis and cardiac involvement	-Myocyte damage is usually not significant near granulomata<br>- Epithelioid granulomata with giant cells, surrounded by lymphocytes and fibrous tissue.<br>-Himmunoistochemstry: CD68+ CD3+<br><br>-Mallory trichrome	https://media.springernature.com/full/springer-static/image/art%3A10.1007%2Fs00428-019-02615-8/MediaObjects/428_2019_2615_Fig2_HTML.jpg?as=webp
**MVMI**			
Fedrigo M, et al. Am J Transplant. 2015 (22)	Case-controlled pilot study evaluated myocardial inflammatory burden Antibody-mediated rejection EMB from 65 right ventricle	-Intravascular monocytes with endothelial damage<br>-Capillary deposition of complement<br><br>-Himmunoistochemstry: C4d + CD3+ intravascular localization	https://ars.els-cdn.com/content/image/1-s2.0-S1600613522000314-gr1.jpg<br>https://ars.els-cdn.com/content/image/1-s2.0-S1600613522000314-gr3.jpg<br>https://ars.els-cdn.com/content/image/1-s2.0-S1600613522000314-gr4.jpg
**TM**			

Leone O, et al. Virchows Arch. 2019 (5)	No data	Early stage: necrotic myocytes myofibre eosinophilic changes<br>vacuolar degeneration<br>coagulative necrosis contraction band necrosis<br>Phase of ‘myocarditis: inflammatory infiltrates primarily composed of macrophages, occasional neutrophils and a small number of lymphocytes CD68+<br>CD3+	https://media.springernature.com/full/springer-static/image/art%3A10.1007%2Fs00428-019-02615-8/MediaObjects/428_2019_2615_Fig7_HTML.jpg?as=webp
**SARS-CoV-2 infection and/or vaccination myocarditis**			
	Many more morphological and biomolecular documentations (about 800 well documented), in the period 2020-2024 due to an increase in autopsies aimed at COVID-19 deaths		

Legend: LM Lymphocytic Myocarditis; CD3+ T lymphocytes; CD68+ macrophages; EMB endomyocardial biopsy; PCR Polymerase Chain Reaction; GCM giant cell myocarditis; CD4+ T Helper lymphocytes; CD8+ cytotoxic T lymphocytes; CD20+ B lymphocytes; CD138+ plasma cells; LHM lympho-histiocitary myocarditis; NM neutrophilic myocarditis; EM eosinophilic myocarditis; Gram stain + histochemical staining to distinguish bacteria; EM eosinophilic myocarditis; CD15+ marker for myeloid lineages and neutrophilic granulocytes; GCM giant cell myocarditis; CS cardiac sarcoidosis; : MVMI myocarditis with vasculitis and microvascular inflammation; C4d + complement component; TM toxic myocarditis
